# Case report: Uncommon gastric metastasis as a presentation of recurrent clear cell renal cell carcinoma

**DOI:** 10.3389/fonc.2024.1354127

**Published:** 2024-05-02

**Authors:** Josep Sabaté-Ortega, Marc Albert-Carrasco, Carmen Escribano-Ferrer, Gerard Grau-Manrubia, Clàudia Fina-Planas, Carme López-Núñez, Eduard Teixidor-Vilà, Elisabet Bujons-Buscarons, Clàudia Montañés-Ferrer, Núria Sala-González

**Affiliations:** ^1^ Department of Medical Oncology, Catalan Institute of Oncology, Doctor Josep Trueta University Hospital, Girona, Spain; ^2^ Precision Oncology Group (OncoGIR-Pro), Girona Biomedical Research Institute (IDIBGI-CERCA), Parc Hospitalari Martí i Julià, Salt, Spain; ^3^ Department of Gastroenterology, Hospital Universitari Doctor Josep Trueta University Hospital, Girona, Spain; ^4^ Department of Pathology, Clínica Girona, Girona, Spain

**Keywords:** renal cell carcinoma, gastric metastasis, prognostic markers, targeted therapy, pathophysiology

## Abstract

Renal cell carcinoma (RCC) is a kidney neoplasm that accounts for 85% of cases and has complex genetic pathways that affect its development and progression. RCC metastasis can occur in 20%-50% of patients and usually affects distant organs. Gastric metastases (GM) from RCC are rare and present as polyp-like growths in the submucosal layer, accounting for 0.2%-0.7% of cases. This case report describes an 84-year-old female with Furhman grade II ccRCC who presented with an atherothrombotic ischemic stroke and gastrointestinal bleeding nine years post-radical nephrectomy. Gastroscopy revealed a 12mm pseudopedicled gastric lesion with ulceration and bleeding, diagnosed as metastatic ccRCC. The discussion focuses on the rarity, diagnostic challenges, and prognostic elements of gastric metastasis from RCC. The median survival after detecting digestive metastasis varies widely, and the mechanisms include direct invasion and dissemination through lymphatic, transcelomic, or hematogenous routes. Prognostic markers encompass patient history, symptoms, time since RCC diagnosis, overall health, and genetic factors. Surgical removal of gastric lesions and targeted therapy are treatment options that can improve survival. This case report highlights the need for further research to enhance diagnostic and treatment strategies for this rare aspect of RCC pathophysiology.

## Introduction

Renal cell carcinoma (RCC) constitutes the majority, approximately 85%, of kidney neoplasms and constitutes a small fraction, approximately 4%, of all adult tumors (1). A younger age at diagnosis is associated with a more aggressive disease phenotype (2). Regarding gender distribution, RCC is more frequently observed in males (3). The five-year survival rates of RCC exhibit significant variation based on the disease stage. They decline from approximately 82 to 84% among individuals diagnosed with stage I RCC to a range of 5.2–6.6% for those presenting with stage IV disease (4). Approximately 2%–3% of all RCC cases have a hereditary origin (5). Typically, RCC displays intricate genetic and molecular changes that propel its development and progression.

RCC involves pivotal pathways in its development and progression. The central HIF pathway is influenced by VHL gene mutations, causing continuous HIF activation, promoting angiogenic factors like VEGF and PDGF, and encouraging tumor growth (6). The PI3K/AKT/mTOR pathway signalling pathway regulates cell growth, differentiation, migration, survival, angiogenesis, and metabolism. Growth factors, hormones, cytokine and many extracellular cues activate PI3K/AKT/mTOR. Dysregulation of this molecular pathway is frequently reported in human cancers including RCC and is associated with aggressive development and poor survival rate (7). The Wnt/β-catenin pathway, affected by VHL loss, contributes to renal tumorigenesis and mTOR pathway activation. This signalling plays a critical role in cancer development by promoting stem cells renwal, cell proliferation, tumor initiation, and metastasis, correlating with poor prognosis and therapy resistance. The mechanism involves Wnt ligands binding to Frizzled receptors and co-receptors, leading to the stabilization of β-catenin, which translocates to the nucleus and regulates target gene expression essential for cancer progression (8, 9). Additionally, its activation enhances epithelial-mesenchymal transition (EMT), facilitating cancer cell invasion and metastasis (10). Recent studies emphasize the pivotal role of hypoxia in driving the EMT pathway, which is integral to metastasis (11). Hypoxia induces EMT by intricately modulating various signaling pathways, including EMT-related transcription factors and microRNA networks. In hereditary papillary RCC, the HGF/MET pathway assumes central importance, particularly influenced by MET gene mutations (12). The activation of HGF/MET initiates downstream signaling cascades, notably involving Ras/MAPK and PI3K/AKT pathways, which significantly contribute to the progression and metastasis of RCC (13). These pathways highlight RCC’s complex nature, where genetic alterations affect multiple signaling cascades.

The majority of relapses tend to occur within a three-year timeframe, but recurrence can manifest unpredictably, sometimes extending beyond 10 years following resection (13). At the initial diagnosis, approximately 8.76% of patients have been reported to experience distal metastasis (DM), with 35.01% of them having multiple metastases (14). In the context of RCC, metastasis has been observed in 20%-50% of patients during follow-up assessments (15). This represents a minority within the spectrum of malignant kidney tumors, accounting for less than 1% of cases (16). Among the various histological variants of RCC, around 30% of clear cell renal cell carcinoma (ccRCC) cases involve metastasis at the time of diagnosis, with the most commonly affected sites being the lungs (45.2%), bones (29.5%), lymph nodes (21.8%), and liver (20.3%) (17). Renal collecting duct renal cell carcinoma (CDCRCC) and papillary renal cell carcinoma (PRCC) generally carry a lower risk of metastasis, with metastatic occurrences primarily observed in distant organs such as the lungs (0.3%), bones (0.3%), liver (0.2%), and lymph nodes (97.6%) (18, 19). In the case of chromophobe RCC, metastasis is observed in 43% of cases in lymph nodes, 31% in bones, 27% in the liver, and 34% in the lungs (20). Unclassified RCC commonly presents with diverse metastatic patterns, encompassing direct infiltration into neighboring organs (42%), bone metastases (52%), adrenal gland involvement (25%), non-regional lymph node metastases (41%) and regional lymph node metastases (52%) (21). It is important to note that instances of gastric metastases originating from RCC are exceedingly rare and are primarily observed in lung, breast, melanoma, and esophageal cancer cases. There is a scarcity of information on gastric metastasis from RCC in the current literature, with most available data being in the form of case reports or literature reviews. As a result, we embarked on an nine-year investigation centered around a case of gastric metastasis of RCC. The objectives of presenting this case report were to reveal the clinical symptoms and characteristics of gastric metastasis, evaluate the prognosis, survival rates, diagnostic methods, and explore the management and treatment options related to gastric metastasis in RCC patients.

## Case presentation

An 84-year-old female with a history of hypertension and diabetes underwent a CT scan in June 2014, initially intended for a vascular study. Remarkably, the scan incidentally revealed a 6.8 cm Furhman grade II ccRCC in the right kidney. Notably, the patient was asymptomatic, without hematuria, pain, or other typical symptoms. Subsequently, she underwent a laparoscopic right radical nephrectomy in June 2014, with the tumor staged as pT1b. Post-surgery, the patient underwent rigorous radiological follow-ups, all of which showed no signs of ccRCC recurrence, indicating a favorable prognosis.

**Figure 1:**
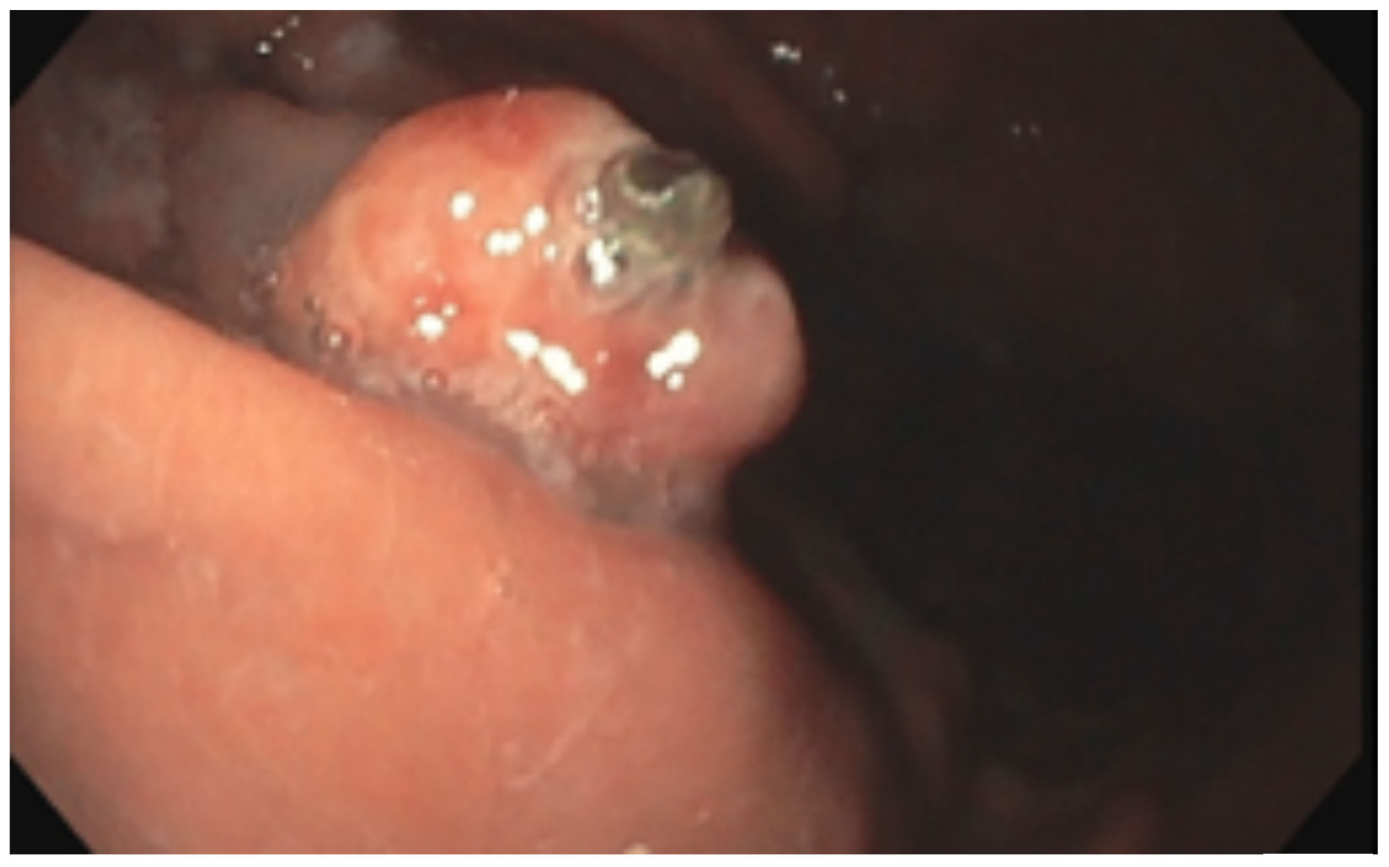
Polypoid formation that 12mm polypoidal lesion on the foot with a pseudopedicled morphology. The lesion’s surface is ulcerated.

In November 2022, nine years after her initial diagnosis and treatment, the patient presented to our center with an atherothrombotic ischemic stroke affecting the left arm. The onset of melenic stools with anemization and without hemodynamic repercussions, prompted an urgent gastroscopy. Gastroscopy revealed a 12mm polypodial lesion with pseudopedicled morphology at the gastric level, presenting ulceration of its surface with endoscopic stigmata of recent bleeding (**Figure 1**). The lesion was excised during the procedure, and hemorrhage control was achieved through the application of mechanical clips and adrenaline injection The patient did not present hemorrhagic recurrence and was discharged from the hospital with outpatient gastroscopy to evaluate endoscopic resection of the gastric lesion. In outpatient gastroscopy at 12 weeks, a gastric polyp described with persistence of an eroded surface with low blood flow was seen. En bloc endoscopic mucosectomy was performed without relevant incidents.

**Figure 2:**
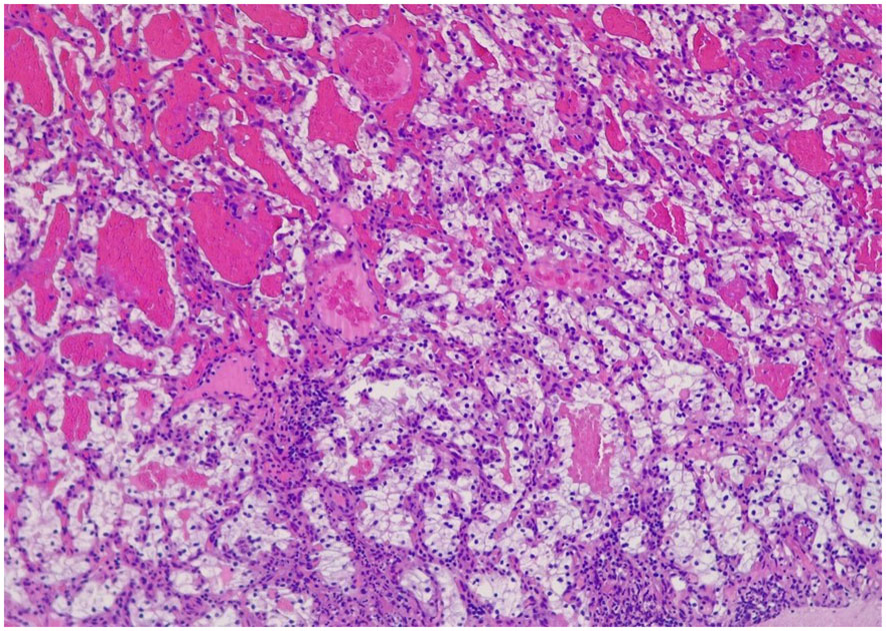
Polypoid formation that presents on the ulcerated surface, in which a tumor can be seen in the lamina propria made up of glands of different sizes, some dilated, which are covered by cells that have large clear cytoplasms and slightly irregular nuclei with small nucleoli eosinophils. They are separated by an edematous stroma.

**Figure 3:**
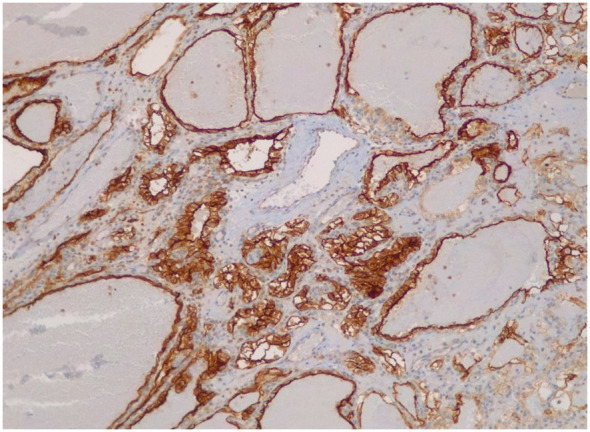
Immunohistochemical staining with CD10 and Keratin CAM5.2 which are positive. The immunohistochemical profile is compatible with clear cell renal carcinoma metastases.

Gastroscopy revealed at the major curve of the gastric body, a 12mm polypodial lesion with pseudopedicled morphology, its surface was ulcerated with blood oozing. In the histological analysis of the gastric polyp, a polypoid formation with an ulcerated surface is described, in which a tumor consisting of cells of different sizes, some dilated, covered by cells with large clear cytoplasms and slightly irregular nuclei, is observed in the lamina propria (**Figure 2**). Immunohistochemistry of the gastric lesion showed positive staining for CD10 and Keratin CAM5.2 (**Figure 3**), consistent with metastatic ccRCC. To rule out metastatic recurrence at other body sites, a CT scan was performed following the gastroscopy. The results demonstrated no evidence of metastatic ccRCC elsewhere in the patient’s body.

En bloc endoscopic mucosectomy was performed without relevant incidents. Following the resection, the patient was closely monitored through regular imaging studies to surveil for any signs of recurrence or progression of the lesion. Notably, the last chest CT scan conducted in June 2023 and abdominal CT scan in July 2023 demonstrated no indications of relapse. Similar findings were observed in laboratory tests, offering a positive outlook for the patient’s ongoing health management and recovery.

## Discussion

The emergence of gastric metastasis signifies an adverse prognostic element, marking a pivotal juncture in the progression of the disease. The median survival after the detection of digestive metastasis is roughly one year, although this timeframe can greatly vary. Metastases from RCC, whether they manifest in the stomach, duodenum, or jejunum, are rare occurrences. Gastric metastases (GM) have been recognized as a location of occurrence in approximately 0.2–0.7% of cases, regardless of the primary tumor’s origin (22).

The survival rates of gastric metastasis in RCC patients vary but generally indicate a poor prognosis. According to a study published in 2021, median patient survival after surgical treatment of gastric metastasis from RCC was 9 months, compared to 6 months without surgical intervention, highlighting the efficacy of surgical intervention (23).The 5-year survival rates for metastasis RCC range from 5 to 30%, indicating the challenging nature of the disease (24). The available literature on RCC metastases in the digestive tract primarily consists of a handful of case reports and literature reviews. Our case report, therefore, stands as one of the few instances that demonstrate comprehension and insight into the infrequent event of RCC metastasis across the digestive tract.

The typical appearance of GM originating from RCC often presents as a polyp-like growth within the submucosal layer, featuring a central depression, as observed in our case. The time elapsed between the initial diagnosis of the primary tumor and the subsequent diagnosis of metastatic tumors can vary depending on the primary tumor’s characteristics. Gastric metastases stemming from lung cancer and malignant melanoma are typically identified within a relatively short timeframe, typically within two years following the primary tumor diagnosis. In contrast, the emergence of gastric metastases from RCC can occur later in the disease progression. RCC tumors tend to exhibit slow growth, follow a sequential pattern of metastasis to other organs, face challenges in adapting to the stomach’s harsh environment, and may possess immunosuppressive properties (25). Additionally, metastases to different parts of the gastrointestinal tract, such as the stomach, duodenum, or other regions, may present with varying clinical symptoms and timelines for detection (26). For instance, metastasis to the duodenum has been reported as occurring after 13 and 27 years (27), whereas gastric metastasis may have a latency period of 7 years (17).

The median time between the onset of RCC and metachronous gastric metastasis was reported to be 8.5 years for solitary metastasis and 5 years for multiple metastasis which extended to 9 years in our particular case (25). The potential mechanisms underlying the gastrointestinal involvement of RCC include direct tumor invasion or dissemination through lymphatic, transcelomic, or hematogenous routes (28). Prognostic and risk markers for gastrointestinal metastasis arising from RCC encompass a spectrum of factors. It includes patient-specific considerations such as a patient’s medical history, especially instances where RCC may have gone undiagnosed, the presence of non-specific symptoms, time since diagnosis, overall health, and poor prognosis during endoscopy. Additionally, tumor genetics, tumor stage, and histological subtype are significant contributors; ccRCC with Von-Hippel VHL gene mutations, Papillary RCC with Type I: MET protooncogene, Type II: SETD2, CKDN2A, and TFE3, and Chromophobe RCC with TERT promoter gene mutations (18, 28–30). These markers are instrumental in assessing the likelihood of metastasis, guiding treatment decisions, and evaluating prognosis.

EMT is correlated with the malignant and invasive characteristics of tumors, and a significant hallmark of EMT in tumor progression involves the reduced expression of E-cadherin (cell adhesion molecule). In gastric cancer, the loss of functional E-cadherin, often due to mutations in the CDH1 gene, disrupts adherens junctions between epithelial cells, leading to decreased cell-cell adhesion. This loss of adhesion facilitates the detachment of cancer cells from the primary tumor mass, allowing them to invade surrounding tissues and enter the bloodstream or lymphatic vessels. Tumor suppression or dysregulation, chromosomal rearrangement, signaling pathway dysregulation (MAPK pathway), and chromosomal rearrangement is the major contributor of gastric metastasis of RCC (31). However, the precise mechanism responsible for the delayed onset of gastric metastasis is yet to determine.

Similar to our case, Yoshida R et al. presented a symptomatic manifestation, comprising anemia, melena as well as transient loss of consciousness in an 85 year old patient. Imaging revealed a hypervascular, lobulated polypoid mass in the middle gastric body and a similar lesion in the pancreas, later confirmed to be metastatic clear RCC through biopsy. The patient was treated with endoscopic resection with no recurrence at 2 years’ follow-up (32). In contrast, Saifi et al. described a 65-year-old male with multi-metastatic ccRCC who developed asympomtoamtic progression to hypervascular gastric metastases despite undergoing eight years of treatment with various chemotherapy and immunotherapy regimens (33). These contrasting cases emphasize the importance of considering individual patient factors, tumor characteristics, and treatment responses in the management of metastatic ccRCC.

In alignment with our study, pertinent literature has featured instances where surgical resection has been elected as a favored therapeutic modality. In a 61-year old man, an 8-year interval was observed between the onset of gastric metastasis and the initial diagnosis of localized right ccRCC (Furhman grade III, classified as PT3An0R0). In this particular case, a laparoscopic wedge resection was initially performed but was later converted to a laparotomy (25). In another case, a 67-year-old man with a history of stage III chronic kidney disease (CKD), a prior ST-elevation myocardial infarction (STEMI), polycystic kidney disease, liver donor renal transplantation, and chronic immunosuppression displayed multiple episodes of melena. Esophagogastrodudenoscopy (EGD) revealed a 2.5 to 3.0 cm popypoid mass in gastric fundus that underwent polypectomy snare and cautery. The procedure was followed by epinephrine injection and hemostasis was achieved (26).

Gastric metastasis following RCC remains a rare phenomenona. Up until 2017, only 37 documented cases of gastric metastasis post RCC were reported, underscoring the rarity of this condition. Out of these 37 cases reported, surgical resection remaind the most commonly performed treatment (28). However, the impact of surgically removing the metastatic gastric tumor on overall survival remains uncertain. The outlook for survival might potentially become more favorable if there are no additional metastatic lesions besides the one in the stomach. Surgical resection could potentially be beneficial in up to 72% of cases involving gastric metastasis with bleeding, as observed in the current case (34). Despite the 27% risks of recurrence, this strategy offers the advantage of postponing systemic therapy initiation, irrespective of whether the resection is performed endoscopically or through surgical means. Additionally, resection proves efficacious and prompt, especially when the metastasis induces acute bleeding. Furthermore, in scenarios involving multiple metastases, complete resection contributes to enhanced patient survival (17, 34).

Alternatively, targeted therapy with tyrosine kinase inhibitors is a viable treatment option, boasting a success rate of up to 75% (35). This approach encompasses the identification of molecular targets within cancer cells and the subsequent development of drugs designed to block or inhibit these specific targets. The efficacy of targeted therapy surpasses that of traditional chemotherapy (CTH), as it precisely targets cancer cells while sparing normal cells, thereby mitigating potential side effects (36). However, it comes with an elevated risk of lesion hemorrhage. In cases involving substantial metastases or active bleeding, it becomes imperative to initiate local hemostatic measures before introducing targeted therapy. The management of RCC with gastric metastasis hinges on factors like tumor stage, the extent of metastasis, and patient-specific characteristics (36).

This case report holds significant scientific importance as it unveils a relatively rare yet crucial aspect of RCC pathophysiology. The exploration of the underlying mechanisms governing the delayed metastasis of RCC to the gastrointestinal tract, particularly the stomach, has the potential to advance the diagnostic and therapeutic strategies employed by healthcare professionals. The prospect of more timely interventions resulting from this understanding has the potential to substantially improve patient prognosis and overall well-being. However, the absence of risk marker evaluation in our patient, hinders the understanding of contributory factors of RCC-linked delayed metastasis to the gastrointestinal tract. This emphasizes the need for further research to improve diagnostic and treatment strategies.

## Conclusion

Gastric metastasis subsequent to RCC represents a rare yet clinically significant event, carrying implications for patient prognosis and therapeutic management. The current study adds valuable insights into this infrequent phenomenon, emphasizing the necessity for meticulous surveillance and prompt intervention in RCC cohorts to ameliorate clinical outcomes. The rarity of gastric metastasis underscores the imperative need for further investigation and empirical case studies to identify risk markers and optimize diagnostic and therapeutic protocols.

## Data availability statement

The original contributions presented in the study are included in the article/supplementary material. Further inquiries can be directed to the corresponding authors.

## Ethics statement

Written informed consent was obtained from the individual(s), and minor(s)’ legal guardian/next of kin, for the publication of any potentially identifiable images or data included in this article.

## Author contributions

JS: Writing – original draft, Writing – review & editing. MA: Writing – review & editing. CE: Writing – review & editing. GG: Writing – review & editing. CF: Writing – review & editing. CL: Writing – review & editing. ET: Writing – review & editing. EB: Writing – review & editing. CM: Writing – review & editing. NS: Writing – review & editing.
